# Acquisition of a Stable and Transferable *bla*_NDM-5_-Positive Plasmid With Low Fitness Cost Leading to Ceftazidime/Avibactam Resistance in KPC-2-Producing *Klebsiella pneumoniae* During Treatment

**DOI:** 10.3389/fcimb.2021.658070

**Published:** 2021-07-20

**Authors:** Jiangqing Huang, Shengcen Zhang, Zhichang Zhao, Min Chen, Yingping Cao, Bin Li

**Affiliations:** ^1^ Department of Clinical Laboratory, Fujian Medical University Union Hospital, Fuzhou, China; ^2^ Department of Pharmacy, Fujian Medical University Union Hospital, Fuzhou, China; ^3^ Department of Laboratory Medicine, Fujian Medical University, Fuzhou, China

**Keywords:** ceftazidime/avibactam, carbapenem-resistant *Klebsiella pneumoniae*, whole-genome sequencing, fitness cost, NDM

## Abstract

The emergence and prevalence of carbapenem-resistant *Enterobacteriaceae* (CRE) have drawn worldwide attention. Ceftazidime/avibactam (CAZ/AVI) gives us a valuable alternative strategy to treat CRE infections. Unfortunately, CAZ/AVI resistance could occur during CAZ/AVI treatment. The CAZ/AVI-resistant Carbapenem-resistant *Klebsiella pneumoniae* (CR-KP) (KP137060) and earlier CAZ/AVI-susceptible isolate (KP135194) from the same hospitalized patient were collected at Fujian Medical University Union Hospital between October and November 2019. In this study, CAZ/AVI MICs of CAZ/AVI-susceptible and -resistant isolates (KP135194 and KP137060) were 4 mg/L and 128 mg/L, respectively; and the two isolates had the same antibiotic resistance pattern to other carbapenems. Two strains were then submitted for whole-genome sequencing and bioinformatic analysis. *ompK36* was not detected in two isolates. No mutation was observed in *bla*_KPC-2_, *ompK35* and *ompK37* in this study and there was no significant difference of the expression in *bla*_KPC-2_, *ompK35* and *ompK37* between the two isolates (*p*>0.05). Two isolates were sequence type 11 and harbored *bla*_KPC-2_, *bla*_SHV-182_ and *bla*_TEM-1B_. Compared with KP135194, KP137060 harbored an additional *bla*_NDM-5_ positive plasmid. *bla*_NDM-5_ gene could be successfully transferred into *E. coli* J53 at a conjugation frequency of 1.14×10^-4^. Plasmid stability testing showed that *bla*_KPC-2_- and *bla*_NDM-5_-harboring plasmids were still stably maintained in the hosts. Growth assay and growth competition experiments showed there was no significant difference in fitness cost between two CR-KP isolates. Our study described the acquisition of a *bla*_NDM-5_-harboring plasmid leading to resistance to ceftazidime/avibactam in KPC-2-producing *Klebsiella pneumoniae* during treatment. This phenomenon deserves further exploration.

## Introduction

The emergence and prevalence of carbapenem-resistant *Enterobacteriaceae* (CRE) have attracted extensive attention (Potter et al., 2016). Carbapenem-resistant *Klebsiella pneumoniae* (CR-KP) is the most common CRE worldwide (Grundmann et al., 2017; Han et al., 2020). CR-KP can lead to many kinds of infections, such as pneumonia, bloodstream infections and urinary tract infections, resulting in high morbidity and mortality (Huang et al., 2018). CR-KP is an extremely severe public health challenge nowadays (Zheng et al., 2017; Jung et al., 2019). Patients with CR-KP infections had few effective treatment options, including polymyxins, tigecycline, aminoglycosides and fosfomycin (Zhang et al., 2019). However, these antibiotics are limited by efficacy and safety (Satlin et al., 2011).

Ceftazidime/avibactam (CAZ/AVI) is a valuable alternative strategy to treat KPC-producing *K. pneumoniae* infections (Tumbarello et al., 2019). CAZ/AVI is a novel combination of ceftazidime and the β-lactamase inhibitor avibactam with activity against class A, class C and some class D carbapenemases, including the *Klebsiella pneumoniae* carbapenemase (KPC) (Zasowski et al., 2015; Shirley, 2018). Furthermore, CAZ/AVI has no activity against class B carbapenemases, such as New Delhi metallo-β-lactamase (NDM) (Thaden et al., 2017; Zhang et al., 2020). NDM from *Klebsiella pneumoniae* was first found in 2009 (Kumarasamy et al., 2010). Up to now, a total of 31 variants (NDM-1 to NDM-31) have been detected globally and the sequences were deposited in NCBI [https://www.ncbi.nlm.nih.gov/pathogens/refgene/#gene_family: (blaNDM)]. Among these variants, NDM-5 was first identified in an *Escherichia coli* strain in the UK in 2011 and has drawn worldwide attention due to its rapid dissemination (Hornsey et al., 2011). Furthermore, NDM-5-producing strains have been usually identified from humans, animals and hospital environments’ sewage water (Hornsey et al., 2011; Yousfi et al., 2015; Parvez and Khan, 2018). Worryingly, NDM-5 has been identified in various species of *Enterobacterales* across many cities in China (Mao et al., 2018; Sun et al., 2018; Guo et al., 2019). Like other variants of NDM, NDM-5 can lead to CAZ/AVI resistance (Wei et al., 2020).

Many studies found that CAZ/AVI resistance could occur during CAZ/AVI treatment, such as mutations in *bla*_KPC_, the increased copy number of the variation of *bla*_KPC_ (*bla*_KPC-3_) and overexpression of *bla*_KPC_ (Shields et al., 2017; Hemarajata and Humphries, 2019; Räisänen et al., 2019; Coppi et al., 2020; Zhang et al., 2020). However, there has been no report of a case of acquiring metallo-β-Lactamase genes during CAZ/AVI treatment. In this study, we reported the emergence of CAZ/AVI-resistance due to acquisition of a metallo-β-lactamase gene during treatment of KPC-2-producing *Klebsiella pneumoniae* infections and identify and validate the reason for CAZ/AVI-resistance emergence using genomic and molecular genetic approaches.

## Materials and Methods

### Bacterial Isolates and Case Report

The CAZ/AVI-resistant CR-KP (KP137060) and earlier CAZ/AVI-susceptible isolate (KP135194) from the same hospitalized patient were collected at Fujian Medical University Union Hospital between October and November 2019. The bacterial species were identified using Vitek-2 (GN cards).

**Figure 1:**
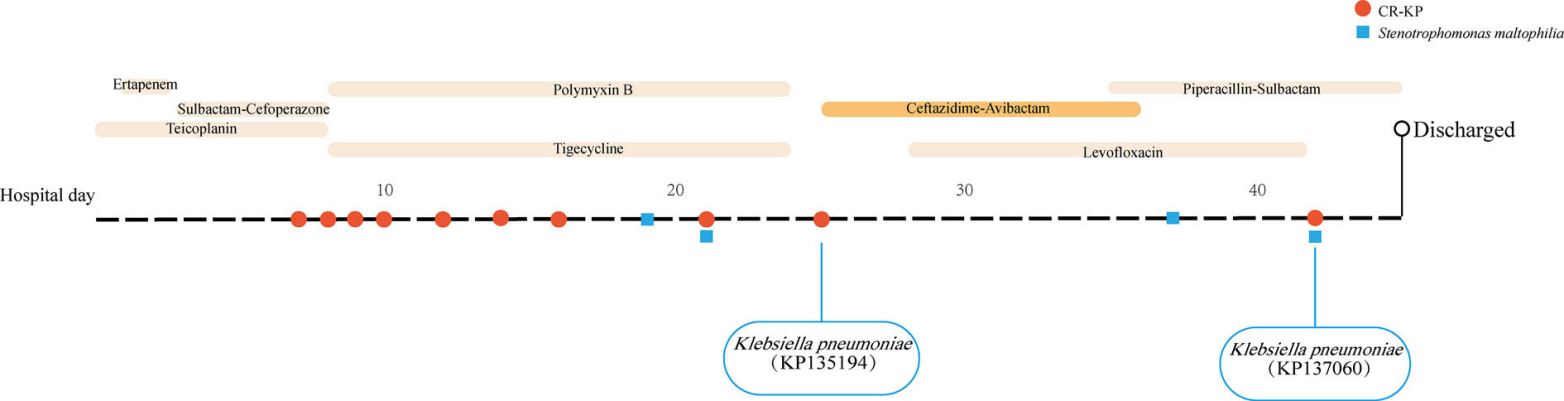
Time courses of infection and treatment of the patient with carbapenem-resistant *K. pneumoniae* infection.

Following a traffic accident, a 67-year-old man was admitted to our ICU with pulmonary infections. Treatment with tigecycline (50 mg every 12 h) and polymyxin B (0.5 miu every 12 h) was initiated after obtaining postbronchoscopic sputum cultures, which yielded CR-KP. However, postbronchoscopic sputum cultures were still positive for CR-KP (KP135194) after 16 days of treatment, susceptible to CAZ/AVI and resistant to carbapenems. Eight CR-KPs have been isolated before KP135194 and they had the same MICs in CAZ/AVI, MEM, ETP and IMP (4µg/ml, ≥16µg/ml, ≥16µg/ml and ≥8µg/ml, respectively). Then the patient was switched to CAZ/AVI treatment. The treatment with CAZ/AVI in monotherapy (2.5 g every 8 h) was administered for 11 days. However, postbronchoscopic sputum cultures were positive for CR-KP (KP137060) again after seven days end of the CAZ/AVI treatment, resistant to both CAZ/AVI and carbapenems. No other CR-KP was isolated between KP135194 and KP137060. *Stenotrophomonas maltophilia* had been recovered in postbronchoscopic sputum cultures on hospital day 19, day 21, day 37 and day 42. The patient was subsequently discharged and turned to another hospital. Microbiologic details, timelines and antibiotic therapies used were summarized in **Figure 1**.

### Antimicrobial Susceptibility Testing

The antimicrobial susceptibility testing was performed by Vitek-2 (Vitek-AST-GN16 and Vitek-AST-GN09) according to the Clinical and Laboratory Standards Institute guidelines (CLSI, 2020). CAZ powder was kindly provided by Pfizer (China) and AVI powder was purchased from GlpBio (Montclair, CA, USA). CAZ/AVI susceptibility testing was performed by agar dilution method. Avibactam was tested at a fixed concentration of 4µg/ml, in combination with 2-fold dilutions of ceftazidime. MICs were interpreted according to CLSI susceptible breakpoint of ≤ 8/4 μg/ml (CLSI, 2020). *E. coli* ATCC 25922 was used as quality control.

### Detection of Carbapenemase Genes and Clonal Relatedness

The presence of carbapenemase genes (*bla*_KPC_, *bla*_NDM_, *bla*_VIM_ and *bla*_IMP_) was identified by PCR as described previously (Li et al., 2014). The complete sequence of *bla*_KPC_ was determined by Sanger sequencing and subsequently submitted to BLAST program (https://blast.ncbi.nlm.nih.gov/Blast.cgi) against the *bla*_KPC_ reference sequence (GenBank accession number: NG_049253.1). The primers of carbapenemase genes are listed in **Table S1**.

Clonal relatedness of two CR-KPs was analyzed by ERIC-PCR as described previously (Smith et al., 2007).

### Expression Analysis of *bla*_KPC-2_, *ompK35* and *ompK37*

Overnight cultures of two CR-KP strains were grown till the logarithmic growth phase in LB broth medium and cells were harvested at an optical density of 1 at 600 nm (OD600). Total RNA was extracted from the two isolates using Trizol reagent (Sigma-Aldrich, USA) according to the manufacturer’s instructions. The expression levels of RNA coding for *bla*_KPC-2_, *ompK35* and *ompK37* were estimated by qRT-PCR using SYBR Green detection reagents (Tiangen, Beijing) on ABI 7500 Real-Time PCR system. The Relative gene expression levels were calculated using the 2^-ΔCT^ formula with the *rpsL* gene as the internal control (Haeili et al., 2017). All samples were performed in triplicate. *rpsL*, *bla*_KPC-2_, *ompK35* and *ompK37* were amplified using primers listed in **Table S1**.

### Conjugation Experiments and Plasmid Stability Testing

Conjugation experiments were performed to test the transferability of *bla*_KPC-2_ and *bla*_NDM-5_-harboring plasmids using filter mating (Shintani et al., 2019). *E. coli* J53 Az^R^ was used as the recipient. The mating mixture was washed from the filter and spread onto MH agar containing sodium azide at 100 mg/liter and imipenem at 1 mg/liter. The grown isolates were selected and identified by PCR (Li et al., 2014). The antimicrobial susceptibility testing of the recipient and transconjugant was performed as described above. The conjugation frequency (CF) was calculated as follow (Shintani et al., 2019):

$$\text{CF}=\frac{\text{Number transconjugants }(\text{CFU}/\text{mL})}{\text{Numbers of donor and recipient cells }(\text{CFU}/\text{mL})}$$

Plasmid stability testing was conducted to evaluate the stability of *bla*_KPC-2_ and *bla*_NDM-5_-harboring plasmids as described previously (Johnson et al., 2016).

### Fitness Cost Assessment

Growth assay and *in vitro* growth competition experiments were tested to assess the fitness cost of KP135194 and KP137060. Growth assay was performed as described previously (Fernández et al., 2012). *In vitro* competition experiments were performed as described previously (Li et al., 2017).

### Whole-Genome Sequencing and Analysis

The genomic DNA samples were extracted from the CAZ/AVI-susceptible and –resistant isolates by TIANamp Bacteria DNA Kit (Tiangen, Beijing), sequenced using the Illumina NovaSeq platform at Shanghai Personal Biotechnology Co., Ltd (Shanghai, China). The raw data were assembled using SPAdesv3.12.0. The genetic relationship of the two strains was determined by average nucleotide identity (ANI) and core genome SNPs analysis (Richter et al., 2016). The Center for Genomic Epidemiology (https://cge.cbs.dtu.dk/services/) was used to identify resistance genes, major outer membrane porin genes (*ompK35*, *ompK36* and *ompK37*) and plasmid types. The multilocus-sequence typing was identified on *MLST*. VFDB (http://www.mgc.ac.cn/VFs/main.htm) was used to predict the virulence genes. The K-type prediction was carried out by Kaptive (http://kaptive.holtlab.net).

For plasmids, the putative extrachromosomal contigs combining the identification with the genes encoding plasmid replication, partition, conjugative transfer or resistance, were recognized as potential plasmid contigs (Botelho et al., 2017; Xu et al., 2018). The *bla*_KPC-2_ and *bla*_NDM-5_ were identified on those plasmid-associated contigs in assembled genomes, determining in silico the *bla*_KPC-2_ (or *bla*_NDM-5_) location on plasmids. The putative plasmid contigs were analyzed and re-assembled to build single circular plasmids by mapping reference complete plasmid genome. Additional sets of PCRs were performed to confirm the circular status of predicted plasmids as reported previously (Carattoli et al., 2018; Xu et al., 2018). Genes were predicted with GeneMarkS™ and further annotated by BLASTP and BLASTN against NR databases and SwissProt. The BLASTN program was used to compare similar plasmids in the international databases.

### Statistical Analysis

Statistical analysis was performed using SAS 9.4. Chi-square test or Fisher’s exact test (two-tailed) or Mann-Whitney test was performed for data comparison. Only *p* < 0.05 was set as the significance level.

## Results

### Clonal Relatedness, Antibiotic Resistances and Carbapenemase Genes

ERIC-PCR analysis demonstrated that two CR-KP isolates were clonally related (**Figure S1**). KP137060 had higher MICs of CAZ/AVI (5-doubling dilution difference) and TGC (1-doubling dilution difference) than KP135194. They had the same MICs in MEM, ETP and IMP. Antimicrobial susceptibility profiles of the two CR-KPs were shown in **Table 1**. *bla*_KPC-2_ was found in both isolates, and *bla*_NDM-5_ was also detected in KP137060.

**Table 1:** Minimal inhibitory concentrations (MICs) of two CR-KP isolates, the *bla*_NDM-5_-positive *E. coli* transconjugant and *E. coli* J53 recipient strains.

Antibiotics (MICs (mg/L)/antimicrobial susceptibility)	Isolates			
	KP135194	KP137060	Transconjugant (*bla*_NDM-5_)	*E. coli* J53
Ampicillin	≥32/R	≥32/R	≥32/R	8/S
Ampicillin/sulbactam	≥32/R	≥32/R	≥32/R	4/S
Piperacillin	≥128/R	≥128/R	≥128/R	≤4/S
Piperacillin/tazobactam	≥128/R	≥128/R	≥128/R	≤4/S
Aztreonam	≥64/R	≥64/R	≤1/S	≤1/S
Cefazolin	≥64/R	≥64/R	≥64/R	≤4/S
Cefuroxime	≥64/R	≥64/R	≥64/R	4/S
Ceftriaxone	≥64/R	≥64/R	≥64/R	≤1/S
Ceftazidime	≥64/R	≥64/R	≥64/R	≤1/S
Imipenem	≥16/R	≥16/R	≥16/R	≤1/S
Meropenem	≥16/R	≥16/R	≥16/R	≤0.25/S
Ertapenem	≥8/R	≥8/R	≥8/R	≤0.5/S
Ceftazidime/avibactam	4/S	128/R	128/R	0.125/S
Ciprofloxacin	≥4/R	≥4/R	≤0.25/S	≤0.25/S
Levofloxacin	≥8/R	≥8/R	≤0.25/S	≤0.25/S
Gentamicin	≥16/R	≥16/R	≤1/S	≤1/S
Amikacin	≥64/R	≥64/R	≤2/S	≤2/S
Tobramycin	≥16/R	≥16/R	≤1/S	≤1/S
Trimethoprim/sulfamethoxazole	≥320/R	≥320/R	≤20/S	≤20/S
Colistin	≤0.5	≤0.5	≤0.5	≤0.5
Tigecycline	2/S	4/I	≤0.5/S	≤0.5/S

### Mutation and Expression Analysis of *bla_KPC-2_*, *ompK35* and *ompK37*

The results of PCR amplification and sequencing of *bla*_KPC-2_ suggested that no mutation was found in *bla*_KPC-2_ in two CR-KP isolates (**Table 2**). We did not detect mutations in *ompK35* and *ompK37* using *ResFinder*, respectively. *ompK36* was not present in two isolates in this study (**Table 2**). Meanwhile, there were no significant differences in the expression of *bla*_KPC-2_, *ompK35* and *ompK37* between the two isolates (*p*=0.2209, *p*=0.4217 and *p*=0.8626, respectively) (**Figure S2**).

**Table 2:** Genetic characteristics and source of two CR-KP isolates.

	Isolate	
	KP135194	KP137060
**Source**	postbronchoscopic sputum	postbronchoscopic sputum
**MLST**	ST11	ST11
***β*-lactamase resistance genes**	*bla_KPC-2_*, *bla_SHV-182_*, *bla_TEM-1B_*, *bla_LAP-2_*	*bla_KPC-2_*, *bla_NDM-5_*, *bla_SHV-182_*, *bla_TEM-1B_*, *bla_LAP-2_*
***bla_KPC-2_* variant**	No mutation	No mutation
***ompK35***	No mutation	No mutation
***ompK36***	-*	–
***ompK37***	No mutation	No mutation
**Other resistance genes**	*rmtB*, *fosA*, *catA2*, *qnrS1*, *sul2*, *tet(A)*, *dfrA14*	*rmtB*, *fosA*, *catA2*, *qnrS1*, *sul2*, *tet(A)*, *dfrA14*
**Plasmid profile**	ColRNAI, IncFII(pHN7A8), IncHI1B, IncR	ColRNAI, IncFII(pHN7A8), IncHI1B, IncR, IncX3
***bla*_KPC-2_ gene location**	IncFII/IncR plasmid (size:146878 bp)	IncFII/IncR plasmid (size:146878 bp)
***bla*_NDM-5_ gene location**	–	IncX3 plasmid (size:46161 bp)
**Hvkp associated genes (K1, K2, rmpA/rmpA2)**	–	–

*Not detected.

### Conjugation Experiments, Plasmid Stability Testing and Fitness Cost Assessment

In this study, the *bla*_NDM-5_ gene was transferred successfully into *E. coli* J53 at a conjugation frequency of 1.14×10^-4^, but *bla*_KPC-2_ failed. The presence of *bla*_KPC-2_ and *bla*_NDM-5_ was confirmed by PCR. The results of antimicrobial susceptibility testing among recipient cell and transconjugant were shown in **Table 1**. Plasmid stability testing showed that *bla*_KPC-2_- and *bla*_NDM-5_-harboring plasmids were still stably maintained in the CR-KP during 150 generations (**Figure S3**).

As shown in **Figure S4**, no significant difference (*p*>0.05) in growth was observed in two CR-KP isolates. The result of *in vitro* growth competition experiments showed that the relative fitness cost was 1.01.

### Whole-Genome Sequencing and Analysis

General genome features of the sequenced genomes of two strains were shown in **Table S2**. Genetic analysis demonstrated that the two CR-KP isolates were sequence type (ST) 11 and harbored beta-lactamase genes. The whole-genome analysis revealed that KP135194 and KP137060 were highly homologous, showing 99.99% ANI values between them. The result of core genome SNPs analysis showed that the number of nonsynonymous SNPs in the core genome was 23 between the two strains. In this study, KP137060 harbored an additional *bla*_NDM-5_. Other resistance genes were detected in both isolates, which were shown in **Table 2**, including *rmtB*, *fosA*, *catA2*, *qnrS1*, *sul2*, *tet (A)* and *dfrA14*. Virulence genes (*rmpA* and *rmpA2*) and capsule types (K1 and K2) genes associated with hypervirulent *K. pneumoniae* (hvKp) were not found (**Table 2**).

**Figure 2:**
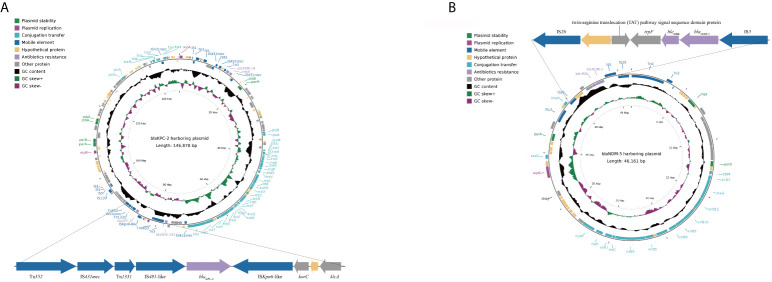
Schematic maps of the *bla*_KPC-2_-harboring plasmid **(A)** and the *bla*_NDM-5_-harboring plasmid **(B)**. Genes are denoted by arrows and colored based on gene function classification. The innermost circle represents GC-Skew [G-C)/G+C]. The black circle represents GC content. The pKPC-CR-hvKP-C789 plasmid was used to reconstruct *bla*_KPC-2_-harboring plasmid as reference plasmid (accession number: CP034417.1). The pCREC-591_4 plasmid was used to reconstruct *bla*_NDM-5_-harboring plasmid as reference plasmid (accession number: CP024825.1).

Compared with the KP135194, KP137060 had an additional plasmid (IncX3) (**Table 2**). Additionally, the results of whole-genome sequencing suggested that *bla*_KPC-2_ (IncFII/IncR) and *bla*_NDM-5_ (IncX3) located in two different plasmids, respectively. The complete nucleotide sequences of *bla*_KPC-2_- and *bla*_NDM-5_-harboring plasmids and sizes of plasmids were shown in **Figure 2**. **Table S3** showed the results of *bla*_NDM-5_-harboring plasmid and its sequences producing significant alignments using the BLAST tool.

For the genetic environment of the *bla*_KPC-2_ **gene, upstream of the *bla*_KPC-2_ gene was located in a Tn*1331* element. Furthermore, a putative IS*Kpn6*-like element was located downstream of the *bla*_KPC-2_ gene, followed by the *korC* gene (a gene encoding transcriptional repressor protein), a gene encoding hypothetical proteins and the *klcA* gene. For *bla*_NDM-5_, upstream of the *bla*_NDM-5_ was located a putative IS*26* element, a gene encoding twin-arginine translocation (TAT) pathway signal sequence domain protein, a gene encoding hypothetical proteins and a *trpF* gene (a gene encoding phosphorybosilanthranilate isomerase gene), were inserted between them. In addition, an IS*5* element was located downstream of *bla*_NDM-5_.

## Discussion

CAZ/AVI has been considered a promising treatment strategy for CRKP infections after approval by the U.S. Food & Drug Administration (FDA) in 2015 (van Duin and Bonomo, 2016). Unfortunately, many reports found CAZ/AVI resistance could occur during CAZ/AVI treatment (Shields et al., 2017; Räisänen et al., 2019; Zhang et al., 2020). According to the rapid risk assessment published by the European Centre for Disease Prevention and Control (ECDC) in 2018, the emergence of CAZ-AVI resistance is an important cross-border threat that should be monitored carefully (Räisänen et al., 2019). Here, in this study, we reported the acquisition of a metallo-β-lactamase gene leading to resistance to ceftazidime/avibactam in KPC-2-producing *K. pneumoniae* during treatment.

CAZ/AVI resistance has been linked to the specific mutations in *bla*_KPC_, *bla*_CTX-M_ and outer membrane protein genes, the increased copy number of the variation of *bla*_KPC_ (*bla*_KPC-3_), overexpression of *bla*_KPC_ and outer membrane protein genes and acquisition of MBL genes (Galani et al., 2019; Hemarajata and Humphries, 2019; Coppi et al., 2020; Zhang et al., 2020). In this study, a MBL gene, *bla*_NDM-5_, was detected in KP137060. Our results indicated that the acquisition of *bla*_NDM-5_ during CAZ/AVI treatment was the possible reason of its resistance to CAZ/AVI. Meanwhile, KP137060 had higher MICs of CAZ/AVI (5-doubling dilution difference) than KP135194 (**Table 1**), which was supported by the fact that CAZ/AVI has no activity against class B carbapenemases (Mueller et al., 2019). In addition, recent study found that the MBL-producing CR-KP isolates, including VIM-producing and VIM/KPC-co-producing isolates, increased after the introduction of CAZ/AVI treatment in an ICU ward (Papadimitriou-Olivgeris et al., 2019). Therefore, our study provided evidence to the fact that widespread CAZ/AVI use might lead to a change in carbapenemases palettes with the substitution of MBL-producing CR-KP isolates for KPC (Papadimitriou-Olivgeris et al., 2019).

In this study, *bla*_KPC-2_ and *bla*_NDM-5_ located in two different plasmids, respectively (**Figure 2**). Our results indicated that *bla*_NDM-5_ was contained on IncX3-type plasmid in KP137060. IncX3 plasmids were usually detected in clinical *bla_NDM-5_*-positive bacteria in China (Cao et al., 2020; Gao et al., 2020). The results of BLAST demonstrated that the *bla*_NDM-5_-harboring plasmid showed >99% sequence identity to the corresponding regions of other plasmids identified in other countries and isolated from different hosts (**Table S3**), suggesting that this type of plasmid was widespread dissemination worldwide. Meanwhile, many studies have characterized the genetic environments of the *bla*_NDM-5_ and various mobile genetic elements played a critical role in the rapid spread of *bla*_NDM-5_ (Pitart et al., 2015; Reynolds et al., 2019). In this study, we described the genetic environment of *bla*_NDM-5_ (**Figure 2**). It is noteworth that such a genetic environment was found in different plasmids in China, probably indicating that they have a common origin (Liu et al., 2019; Cao et al., 2020). Additionally, the genetic environment of *bla*_NDM-5_ was similar to those in previously reported clinical *K. pneumoniae* isolates in India and Spain (Krishnaraju et al., 2015; Pitart et al., 2015). The results of this study suggested that the genetic environment of *bla*_NDM-5_ **could contribute to the widespread of CAZ/AVI-resistant CR-KPs.

In addition, our study demonstrated that the *bla*_NDM-5_-harboring plasmid was transferrable and stable. Given the high transferability and stability of *bla*_NDM-5_-harboring plasmid in clinical CR-KP, the spread of *bla*_NDM-5_-harboring plasmids into other *K. pneumoniae* had the potential to cause refractory infections due to limited therapeutic options. Futhermore, there were no significant fitness cost compared with KP135194 in this study (**Figure S4**), indicating that no considerable fitness costs were identified in carriage of the plasmid containing *bla*_NDM-5_ could be accepted as an important advantage of CAZ/AVI-resistant CR-KP clonal propagation, increasing the risk of dissemination.

In the present case, the patient was treated with 11 days of CAZ/AVI (2.5 g; administered intravenously every 8 h) treatment. After that, CAZ/AVI-resistant CR-KP was identified and clonally related to earlier CAZ/AVI-susceptible CR-KP. Risk factors associated with acquired CR-KP colonization included previous exposure to tigecycline or β-lactam/β-lactamases inhibitor, ICU stays and invasive processes or surgical operations (Qin et al., 2020). In current study, the patient stayed in ICU for up to 30 days and received tigecycline treatment before CAZ/AVI treatment. CR-KP was not isolated when the patient was admitted to the hospital. Additionally, CR-KP was most common among CRE in our hospital (He et al., 2016; Li et al., 2019). Based on the above-mention facts, we speculated the patient might get CR-KP colonization during his stay at ICU and acquisition of a *bla*_NDM-5_-harboring plasmid in KP137096 was caused mainly by the selective pressure of CAZ/AVI usage.

There were some limitations to our study. First, the CRE-screening tests were not carried out to exclude the presence of the NDM-5 producer as colonizer using specimens from sputum or/and rectal swabs. Meanwhile, because the determination was not performed to detect the carbapenem genes in clinical CRE strains in our hospital, we could not know the origin of *bla*_NDM-5_. Second, the CAZ/AVI-resistant CR-KP strain was isolated after CAZ/AVI treatment. Therefore, we could not exclude the probability that KPC-2-producing *K.pneumonia* has harbored the *bla*_NDM-5_ from different bacteria that carried it through HGT during the patient’s hospitalization, rather than the CAZ-AVI treatment. Third, we only used additional PCR sets to confirm the circular status of predicted plasmids in this study. The results of plasmid contents should be more reliable if the S1-PFGE coupled with hybridization could be used in this study.

## Conclusions

Our study demonstrated the acquisition of a *bla*_NDM-5_-harboring plasmid leading to resistance to ceftazidime/avibactam in KPC-2-producing *Klebsiella pneumoniae* during treatment. Meanwhile, the *bla*_NDM-5_-harboring plasmid was transferrable and the conjugation frequency was 1.14×10^-4^. Furthermore, no considerable fitness costs were identified in the carriage of the plasmid containing *bla*_NDM-5_. Therefore, careful monitoring of resistance development by bacterial cultures and subsequent susceptibility testing is significant. Continued surveillance is required to avoid CAZ/AVI-resistance CR-KP dissemination.

## Data Availability Statement

The datasets presented in this study can be found in online repositories. The complete genome sequence of CR-KP strains KP135194 and KP137060 were deposited in GenBank with accession numbers JABJTP000000000 and JABJWR000000000 under BioProject PRJNA633438. The complete sequences of *bla*_NDM-5_-harboring plasmid and *bla*_KPC-2_-harboring plasmid were submitted to GenBank under accession numbers MW218142 and MW218143, respectively.

## Ethics Statement

The studies involving human participants were reviewed and approved by the Medical Ethics Committee of Fujian Medical University Union Hospital (No.2020KY088). The patients/participants provided their written informed consent to participate in this study. Written informed consent was obtained from the individual(s) for the publication of any potentially identifiable images or data included in this article.

## Author Contributions

JH and SZ performed experiments. JH and ZZ designed the study and drafted the manuscript. SZ analyzed the data. YC and MC supervised the study. BL revised the manuscript critically for important intellectual content. All authors contributed to the article and approved the submitted version.

## Funding

This work was supported by the Joint Funds for the innovation of science and Technology, Fujian province (Grant number: 2017Y9049, 2017Y9051) and the Educational and Scientific Research Project for Young and Middle-Aged Teachers of Fujian Province (Grant number: JAT190191).

## Conflict of Interest

The authors declare that the research was conducted in the absence of any commercial or financial relationships that could be construed as a potential conflict of interest.
