# Supplementary material for: Acquisition of a Stable and Transferable bla NDM-5-Positive Plasmid With Low Fitness Cost Leading to Ceftazidime/Avibactam Resistance in KPC-2-Producing Klebsiella pneumoniae During Treatment
Source: Front Cell Infect Microbiol. 2021 Jul 20;11:658070. doi: 10.3389/fcimb.2021.658070 (PMC8329419; doi:10.3389/fcimb.2021.658070)
Supplement: Supplementary file 1 [file DataSheet_1.docx]

**Supplemental material**

**Acquisition of a stable and transferable *bla*_NDM-5_-positive plasmid with low fitness cost leading to ceftazidime/avibactam resistance in KPC-2-producing *Klebsiella pneumoniae* during treatment**

Jiangqing Huang^1^, Shengcen Zhang^1^, Zhichang Zhao^2^, Min Chen^3^, Yingping Cao^1,*^, Bin Li^1,*^

^1^ *Department of Clinical Laboratory, Fujian Medical University Union Hospital,* *Fuzhou, Fujian, 350001, China*

^2^ *Department of Pharmacy, Fujian Medical University Union Hospital, Fuzhou, Fujian, 350001, China*

^3^ *Department of Laboratory Medicine, Fujian Medical University, Fuzhou, Fujian, 350001, China*

**^*^ Correspondence**

Prof. Yingping Cao

Department of Clinical Laboratory, Fujian Medical University Union Hospital, 29 Xinquan Rd., Fuzhou, Fujian, 350001. China;

E-mail: caoyingping@aliyun.com

Prof. Bin Li

Department of Clinical Laboratory, Fujian Medical University Union Hospital, 29 Xinquan Rd., Fuzhou, Fujian, 350001. China;

E-mail: [leonlee307@hotmail.com](mailto:leonlee307@hotmail.com)

**Table**

Table S1.Nucleotide sequences of primers used in this study

| Primer name | Sequence (5' to 3' ) | Size of product (bp) | Reference |
| --- | --- | --- | --- |
| **PCR** |  |  |  |
| *bla*_KPC_-F | GCTACACCTAGCTCCACCTTC | 709 | (Wolter et al., 2009) |
| *bla*_KPC_-R | GCATGGATTACCAACCACTGT |  |  |
| *bla*_NDM_-F | ACCGCCTGGACCGATGACCA | 263 | (Shahcheraghi et al., 2013) |
| *bla*_NDM_-R | GCCAAAGTTGGGCGCGGTTG |  |  |
| *bla*_VIM_-F | ATGGTGTTTGGTCGCATATC | 510 | (Xiong et al., 2006) |
| *bla*_VIM_-R | TGGGCCATTCAGCCAGATC |  |  |
| *bla*_IMP_-F | CTACCGCAGCAGAGTCTTTG | 587 | (Woodford et al., 2004) |
| *bla*_IMP_-R | AACCAGTTTTGCCTTACCAT |  |  |
| *bla*_KPC_ (complete sequence)-F | GCTACACCTAGCTCCACCTTC | 968 | (Wozniak et al., 2019) |
| *bla*_KPC_ (complete sequence)-R | ACAGTGGTTGGTAATCCATGC |  |  |
|  |  |  |  |
| **RT-PCR** |  |  |  |
| *bla*_KPC_-F | CAGCTCATTCAAGGGCTTTC | 196 | (Subirats et al., 2017) |
| *bla*_KPC_-R | GGCGGCGTTATCACTGTATT |  |  |
| *ompK35*-F | CGCAATATTCTGGCAGTGGTG | 165 | This study |
| *ompK35*-R | ATAGGTGGTATCGTCGCTGC |  |  |
| *ompK37*-F | CAACAACACCGAGACCTCCA | 171 | This study |
| *ompK37*-R | GCATAGGTGTAGGAGTCGCC |  |  |
| *rpls*-F | CCGTGGCGGTCGTGTTAAAGA | 109 | (Haeili et al., 2017) |
| *rpls*-R | GCCGTACTTGGAGCGAGCCTG |  |  |

Table S2. General genome features of the sequenced genomes

|  |  | Isolate | |
| --- | --- | --- | --- |
|  |  | KP135194 | KP137060 |
|  | Size (bp) | 5,657,667 | 5,702,404 |
|  | GC content (%) | 57 | 56.91 |
|  | N50 (bp) | 137,184 | 129,409 |
|  | L50 (bp) | 14 | 15 |
|  | No. of subsystems | 397 | 398 |
|  | No. of coding sequences | 112 | 116 |
|  | No. of genes | 5480 | 5534 |
|  | No. of pseudogenes | 105 | 107 |
|  | No. of RNAs | 72 | 71 |
|  | No. of rRNAs (5S, 16S, 23S) | 2, 1, 1 | 2, 1, 1 |
|  | No. of tRNAs | 68 | 67 |
|  | No. of CRISPR arrays | 1 | 1 |
| No. of genes in Database (%) | |  |  |
|  | NR | 98.9051 | 98.8977 |
|  | eggNOG | 90.8029 | 90.4409 |
|  | KEGG | 61.5146 | 60.9866 |
|  | Swiss-Prot | 82.8102 | 82.4359 |
|  | GO | 75.9854 | 75.6596 |
| BioSample | | SAMN14943954 | SAMN14944066 |

Table S3. The results of *bla*_NDM-5_-harboring plasmid and its sequences producing significant alignments using BLAST tool

|  | Description | Max Score | Total Score | Query Cover | E value | Per. Ident | Accession | Origin | Year | Host |
| --- | --- | --- | --- | --- | --- | --- | --- | --- | --- | --- |
| 1 | *Escherichia coli* strain KW53T plasmid pKW53T-NDM, complete sequence | 85227 | 86092 | 100% | 0 | 99.99% | KX214669.1 | United Arab Emirates | 2017 | Human |
| 2 | *Escherichia coli* strain Esco-5256cz plasmid pEsco-5256cz, complete sequence | 85216 | 86081 | 100% | 0 | 99.99% | MG252891.1 | Czech | 2017 | / |
| 3 | *Klebsiella pneumoniae* strain SCKLB138 plasmid pSCKLB138-2, complete sequence | 85079 | 86093 | 100% | 0 | 99.99% | MH161191.1 | China | 2018 | / |
| 4 | *Escherichia coli* plasmid pBJ114-46, complete sequence | 84874 | 86097 | 100% | 0 | 99.99% | MF679143.1 | China | 2018 | Human |
| 5 | *Escherichia coli* strain CREC-591 plasmid pCREC-591_4, complete sequence | 84703 | 86119 | 100% | 0 | 100.00% | CP024825.1 | Korea | 2017 | Human |
| 6 | *Escherichia coli* strain L41-1 plasmid pL41-1-4 | 84394 | 85914 | 100% | 0 | 99.93% | CP034730.1 | China | 2018 | Animals |
| 7 | *Morganella morganii* strain L241 plasmid pNDM5-L241, complete sequence | 83554 | 86113 | 100% | 0 | 100.00% | CP033057.1 | China | 2018 | Human |
| 8 | *Escherichia coli* strain OM26 plasmid pOM26-1, complete sequence | 83044 | 84176 | 97% | 0 | 99.99% | KP776609.1 | United Arab Emirates | 2015 | / |
| 9 | *Escherichia coli* strain VH1 plasmid pVH1, complete sequence | 82784 | 86119 | 100% | 0 | 100.00% | CP028705.1 | China | 2017 | Environment |
| 10 | *Klebsiella pneumoniae* strain SCKLB684 plasmid pSCKLB684-ndm, complete sequence | 82773 | 86108 | 100% | 0 | 99.99% | MH781720.1 | China | 2018 | / |

Figure

Figure legend

Figure S1. ERIC profiles of two CR-KPs in this study.

Figure S2. Relative *bla*_KPC-2_, *ompK35* and *ompK37* expression level in two CR-KPs.

Figure S3. High stability of *bla*_KPC-2_- and *bla*_NDM-5_-harboring plasmids in KP137060 and *bla*_KPC-2_-harboring plasmid in KP135194.

Figure S4. Growth curves of KP135194 and KP137060.

Figure S1


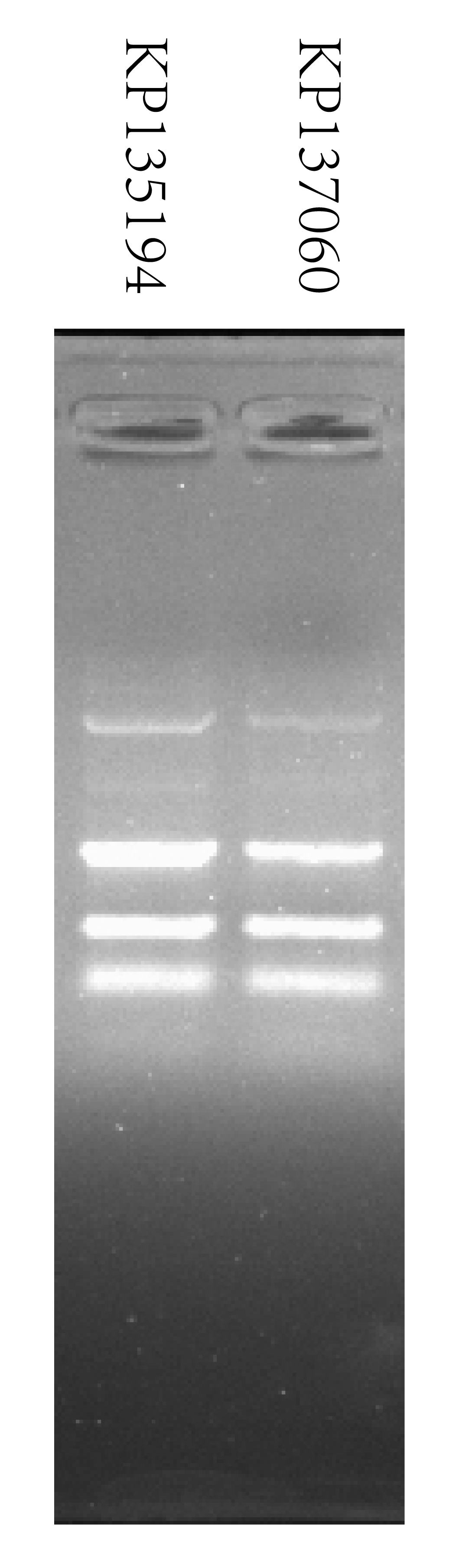


Figure S2


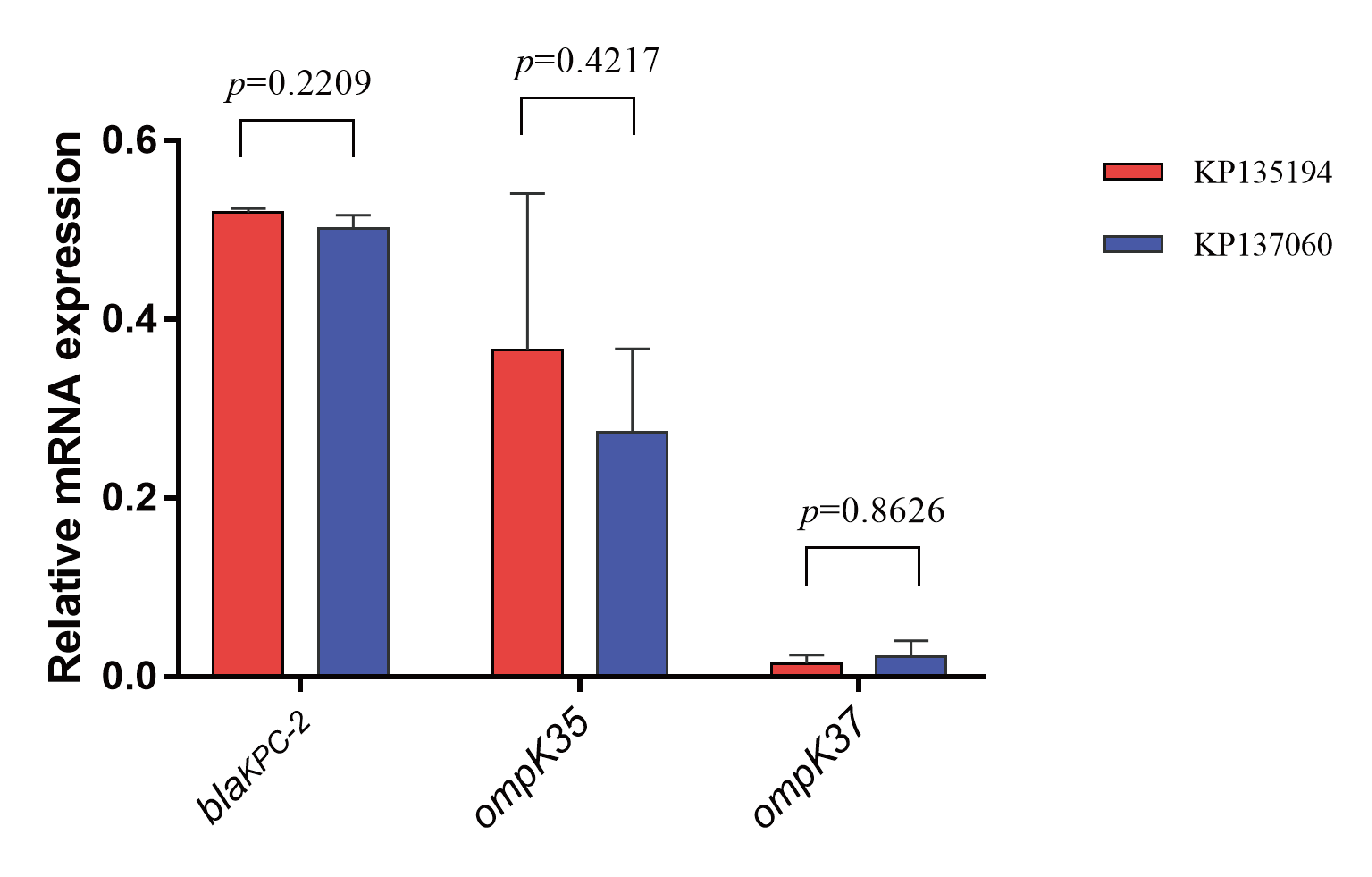


Figure S3


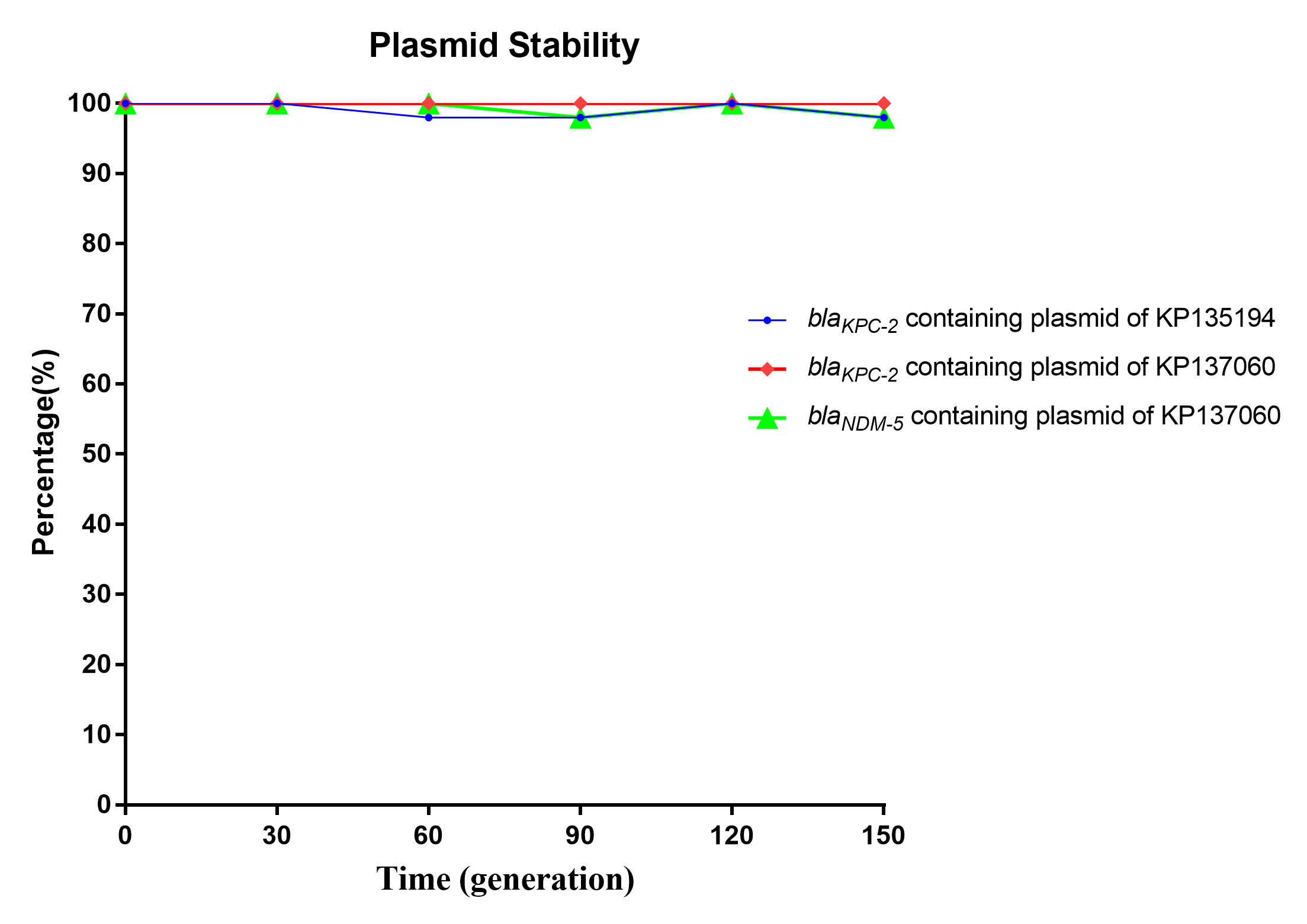


Figure S4


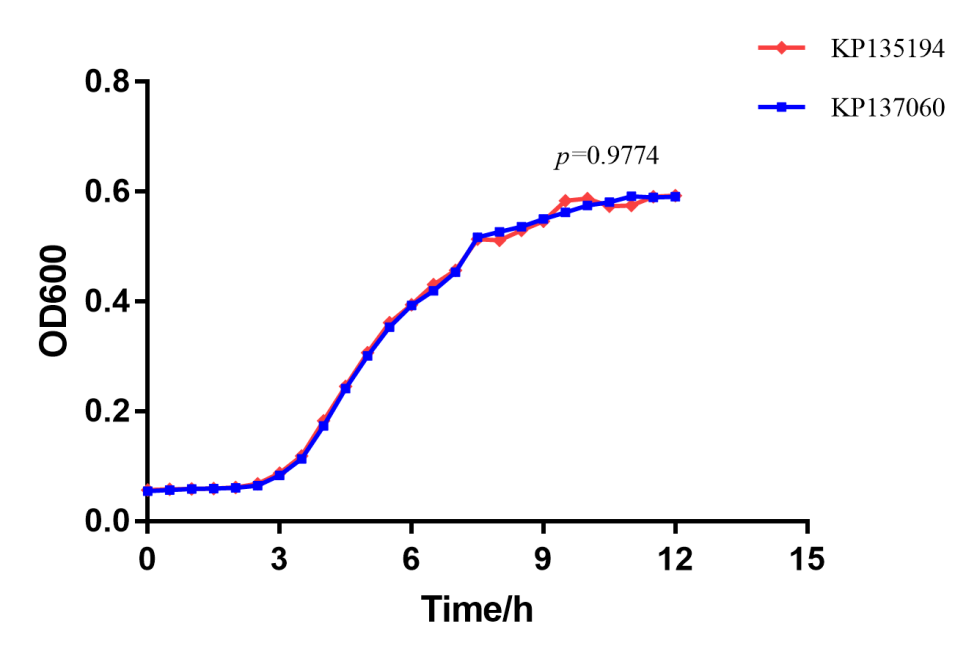


**Reference**

Haeili, M., Javani, A., Moradi, J., Jafari, Z., Feizabadi, M.M., and Babaei, E. (2017). MgrB alterations mediate colistin resistance in *Klebsiella pneumoniae* isolates from Iran. *Front Microbiol* 8**,** 2470. doi: 10.3389/fmicb.2017.02470.

Shahcheraghi, F., Nobari, S., Rahmati Ghezelgeh, F., Nasiri, S., Owlia, P., Nikbin, V.S., et al. (2013). First report of New Delhi metallo-beta-lactamase-1-producing *Klebsiella pneumoniae* in Iran. *Microb Drug Resist* 19(1)**,** 30-36. doi: 10.1089/mdr.2012.0078.

Subirats, J., Royo, E., Balcázar, J.L., and Borrego, C.M. (2017). Real-time PCR assays for the detection and quantification of carbapenemase genes (*bla*_KPC_, *bla*_NDM_, and *bla*_OXA-48_) in environmental samples. *Environ Sci Pollut Res Int* 24(7)**,** 6710-6714. doi: 10.1007/s11356-017-8426-6.

Wolter, D.J., Kurpiel, P.M., Woodford, N., Palepou, M.F., Goering, R.V., and Hanson, N.D. (2009). Phenotypic and enzymatic comparative analysis of the novel KPC variant KPC-5 and its evolutionary variants, KPC-2 and KPC-4. *Antimicrob Agents Chemother* 53(2)**,** 557-562. doi: 10.1128/aac.00734-08.

Woodford, N., Tierno, P.M., Jr., Young, K., Tysall, L., Palepou, M.F., Ward, E., et al. (2004). Outbreak of *Klebsiella pneumoniae* producing a new carbapenem-hydrolyzing class A beta-lactamase, KPC-3, in a New York Medical Center. *Antimicrob Agents Chemother* 48(12)**,** 4793-4799. doi: 10.1128/aac.48.12.4793-4799.2004.

Wozniak, A., Paillavil, B., Legarraga, P., Zumarán, C., Prado, S., and García, P. (2019). Evaluation of a rapid immunochromatographic test for detection of KPC in clinical isolates of *Enterobacteriaceae* and *Pseudomonas* species. *Diagn Microbiol Infect Dis* 95(2)**,** 131-133. doi: 10.1016/j.diagmicrobio.2019.05.009.

Xiong, J., Hynes, M.F., Ye, H., Chen, H., Yang, Y., M'Zali, F., et al. (2006). *bla*_IMP-9_ and its association with large plasmids carried by *Pseudomonas aeruginosa* isolates from the People's Republic of China. *Antimicrob Agents Chemother* 50(1)**,** 355-358. doi: 10.1128/aac.50.1.355-358.2006.
